# The Relationship between Serum 25-Hydroxy Vitamin D and Insulin Sensitivity and  ***β***-Cell Function in Newly Diagnosed Type 2 Diabetes

**DOI:** 10.1155/2015/636891

**Published:** 2015-02-08

**Authors:** Yuan Gao, Xinchi Wu, Qi Fu, Yanyun Li, Tao Yang, Wei Tang

**Affiliations:** ^1^Department of Endocrinology, The Affiliated Jiangyin Hospital of Southeast University Medical College, 163 Shoushan Road, Jiangyin 214400, China; ^2^Department of Endocrinology, The First Affiliated Hospital of Nanjing Medical University, 300 Guangzhou Road, Nanjing 210036, China

## Abstract

The aim of this study was to investigate the relationship between serum 25-hydroxy vitamin D (25-OHD) and insulin sensitivity and *β*-cell function in newly diagnosed type 2 diabetes. 395 newly diagnosed type 2 diabetes patients were enrolled in this study. Venous blood samples were collected at 0 min, 30 min, and 120 min of OGTT to measure serum glucose and insulin. Matsuda ISI and HOMA-IR were used to determine insulin sensitivity. The ratio of 0–120 min area under curve of insulin to glucose (insulin release index, INSR) was calculated as surrogate index of *β*-cell insulin secretion function. The products of insulin secretion indices multiplied by Matsuda insulin sensitivity index were used as disposition indices. Patients were divided into three groups according to tertiles (T1, T2, and T3) of 25-OHD concentration. There was significant difference among three groups for HOMA-IR, Matsuda ISI, and INSR. HOMA-IR, Matsuda ISI, INSR, and DI were undifferentiated among three groups in male patients. But HOMA-IR, Matsuda ISI, and INSR were significantly different among three groups in female patients after being adjusted by confounding factors. In conclusion, serum 25-OHD is associated with insulin sensitivity and *β*-cell function for female newly diagnosed type 2 diabetes patients, and the association is ambiguous in males.

## 1. Introduction

Accumulated evidences indicated that abnormal vitamin D status is associated with the etiology of type 2 diabetes. Compared with subjects with normal glucose tolerance, patients with type 2 diabetes and impaired glucose tolerance (IGT) have lower vitamin D levels [1, 2]. Some prospective studies suggested vitamin D deficiency increases the risk of type 2 diabetes [3, 4]. However, the specific mechanisms by which vitamin D influences the risk of type 2 diabetes are not very clear. Insulin resistance and deteriorated *β*-cell function are the two major pathophysiologic aspects of type 2 diabetes. Several surveys reported significant association of vitamin D with insulin resistance and *β*-cell dysfunction. But not all studies obtained concordant conclusion. Nonetheless, most of these surveys evaluated insulin sensitivity and *β*-cell function using indices just calculated from fasting glucose and insulin, which is not fairly accurate [5, 6]. Therefore, the aim of this study was to investigate the relationship between serum 25-hydroxy vitamin D (25-OHD) and insulin sensitivity, *β*-cell function in newly diagnosed type 2 diabetes, using more predominant evaluation indicators of insulin resistance and *β*-cell function which were derived from oral glucose tolerance (OGTT).

## 2. Research Design and Methods

### 2.1. Subjects

All subjects in this study came from a community survey of urban population over 40 years who lived in Gulou district, Nanjing city, China, from September to October 2012. Subjects with severe pancreatic disease, liver disease, and renal disease and those with excessive fasting blood glucose were excluded. According to the criteria of World Health Organization (WHO) 1999, 395 patients with newly diagnosed diabetes were enrolled in this study, including 157 men and 238 women, with average age of 59.62 ± 8.15 years. The study was approved by the ethics committee of the First Affiliated Hospital of Nanjing Medical University. Verbal informed consent was obtained from all participants.

### 2.2. Measurements

The height, weight, systolic blood pressure (SBP), and diastolic blood pressure (DBP) were measured by trained doctors or nurses. Body mass index (BMI) was calculated by dividing weight in kilograms (kg) by height in squared meter (m^2^). Blood pressure and pulse were taken as the mean of 3 consecutive measurements after at least 5 minutes rest.

After 10–12 hours overnight fasting, venous blood samples were collected to measure fasting plasma glucose (FPG), fasting serum insulin (INS0), high density lipoprotein cholesterol (HDL), low density lipoprotein cholesterol (LDL), triglyceride (TG), alanine aminotransferase (ALT), and *γ*-glutamyltransferase (GGT). Then the 75 g oral glucose tolerance tests (OGTT) were performed, and venous blood samples were obtained at 30 and 120 minutes after glucose load for measuring the plasma glucose (30 min plasma glucose, PG30, and 120 min plasma glucose PG120) and serum insulin (30 min serum insulin, INS30, and 120 min serum insulin, INS120). Glycated hemoglobin (HbA1c) was examined with capillary blood (Variant II, Bio-Rad). Blood glucose was measured by hexokinase method. HDL, LDL, TG, ALT, and GGT were measured using chemiluminescence methods on the autoanalyzer (Modular E170, Roche). Serum insulin was measured by radioimmunoassay (Iodine [^125^I] Insulin Radioimmunoassay Kit, Beijing North Institute of Biological Technology), and the coefficient variation of intra-assay and interassay was <10%. Serum 25-OHD was measured by enzyme immunoassay (25-Hydroxy Vitamin D Kit, Immunodiagnostic Systems Limited, IDS Ltd.), with coefficient variation of intra-assay <8% and interassay <10%.

### 2.3. Calculations

Insulin resistance of liver was estimated using the homeostasis model assessment (HOMA-IR), which was calculated as follows: HOMA-IR = insulin (mIU/L) × glucose (mmol/L)/22.5 [7]. Matsuda insulin sensitivity index (Matsuda ISI) was used to evaluate whole-body insulin sensitivity, which was calculated as $10000/\sqrt{\left(G0\times I0\right)\times \left(\bar{G}\times \bar{I}\right)}$, where $\bar{G}$ and $\bar{I}$ are the average levels of plasma glucose in mg/dL and insulin in mIU/L of OGTT [8].

To evaluate the insulin secretion, the area under insulin (AUCins) and glucose curves (AUCglu) during OGTT were calculated, and the ratio of AUCins to AUCglu was a surrogate index for insulin release (which was defined as INSR) [9]. Disposition Index (DI, calculated as Matsuda ISI × INSR) was used to assess *β*-cell function, which combines both insulin secretion and insulin sensitivity [10].

### 2.4. Statistical Analysis

According to the tertiles of 25-OHD concentration in total population, the subjects were divided into three groups (low tertile T1, middle tertile T2, and high tertile T3). One way-ANOVA and the chi-square test were used to analyze the differences among groups. Univariate analysis was used to adjust for confounding factors. All statistical analyses were carried out using the Statistical Package for Social Science for Windows (SPSS, Version 13.0).

## 3. Results

The baseline characteristics of three groups were shown in Table 1. The average 25-OHD concentration was 29.15 ± 6.15, 42.49 ± 3.78, and 59.83 ± 8.17 nmol/L in T1, T2, and T3 groups, respectively. TG, TC, and the sex ratio had significant difference among three groups. Compared with T1 group, subjects in T3 group had lower TG and TC levels, whereas there was no significant difference of age, BMI, blood pressure, HDL, LDL, ALT, GGT, and HbA1c among three groups.

In total subjects, there was significant difference of HOMA-IR, Matsuda ISI, and INSR among three groups. As the level of vitamin D increased, Matsuda ISI had a rising trend. On the contrary, HOMA-IR and INSR gradually declined (shown in Table 2). After adjustment for sex, age, BMI, and blood pressure, the difference of HOMA-IR and INSR among groups disappeared, whereas the difference of Matsuda ISI among three tertiles was still significant. Additionally, after further adjustment for TG, TC, HDL, LDL, ALT, GGT, and HbA1c, there was no significant difference of HOMA-IR, Matsuda ISI, and INSR among three vitamin D tertiles. DI was not different among three groups before or after adjustment for confounding factors.

Table 3 showed the insulin sensitivity and *β*-cell function of vitamin D tertiles in female and male subjects. In males, no significant difference of HOMA-IR, Matsuda ISI, INSR, and DI was found among three groups before or after adjustment for confounding factors. In females, there was significant difference of HOMA-IR, Matsuda ISI, and INSR among three vitamin D tertiles, even though adjusting for sex, age, BMI, blood pressure, TG, TC, HDL, LDL, ALT, GGT, and HbA1c the difference was still significant. With the increasing level of vitamin D of females, Matsuda ISI trended to increase; however, HOMA-IR and INSR tended to decrease.

## 4. Discussion

In recent years, the influence of vitamin D on diabetes becomes a research hotpot; many studies paid attention to the relationship between vitamin D and insulin sensitivity and *β*-cell function [11]. In the present study, serum 25-OHD showed positive association with Matsuda ISI and negative association with HOMA-IR. After adjustment for metabolic confounding factors including sex, age, BMI, blood pressure, and serum lipid, the association attenuated. The influence of obesity and dyslipidemia on insulin resistance may contribute to this phenomenon. The association between vitamin D and insulin resistance may be diluted by the intense association obesity and insulin resistance. Some researches demonstrated that excessive fat accumulation could reduce the concentration and activity of the vitamin D in adipose tissue [12]. Vitamin D also affects obesity; low serum vitamin D levels always accompany increased serum parathyroid hormone levels and calcium influx of fat cells, which stimulates lipogenesis, inhibits fat decomposition, and aggravates obesity [13].

Some recent studies of molecular biology had indicated that vitamin D may influence insulin sensitivity in more than one way. In target cells of insulin action, vitamin D could increase insulin receptor, enhance the activity of transcription factors, and regulate the cell calcium concentration; all these effects can improve insulin sensitivity [14, 15]. Inflammation is one of the important physiopathologic mechanisms. Vitamin D could regulate the immunological reaction of macrophages and monocytes, reduce the concentration of interleukin-1 (IL-1), interleukin-6 (IL-6), and tumor necrosis factor-*α* (TNF-*α*), and so on, and thereby relieve inflammatory reaction [16]. On the contrary, vitamin D deficiency is associated with high level of inflammatory factors such as IL-6, TNF-*α*, and C-reactive protein (CRP) [17, 18].

There are also several researches that studied the interaction between vitamin D and *β*-cell function. *β*-cells express vitamin D receptor, which can bind to 1,25-dihydroxyvitamin D. There is vitamin D acting element in insulin gene promoter region, and vitamin D can activate the transcription of insulin gene [14, 19]. These studies suggested that vitamin D promotes insulin synthesis and secretion. Meanwhile, *β*-cells express 25-OHD-1 alpha-hydroxylase (CYP27B1), which could facilitate vitamin D activation. Vitamin D can also regulate *β*-cell calcium concentration and influence insulin secretion through calcium-dependent pathway [20]. In our study, insulin secretion index INSR was different among three vitamin D tertiles; however, the difference attenuated after adjustment for sex, age, BMI, blood pressure, and so on. In females, INSR was different among three vitamin D tertiles although adjusted for metabolic confounding factors. Interestingly, among three vitamin D tertiles, INSR was the lowest in higher tertile. This phenomenon may be attributed to the implication of “INSR,” which only represents the secretion function of *β*-cell regardless of the influence of insulin resistance. During pathophysiology of type 2 diabetes, as insulin resistance developed, *β*-cells compensatorily secret more insulin. When *β*-cell fails to compensate, type 2 diabetes occurs. The subjects in our study were all newly diagnosed type 2 diabetes, whose *β*-cell was in early phase of decompensation. Subjects with higher vitamin D had better insulin sensitivity, so their *β*-cell needed to secret less insulin; this may explain the reason why INSR was the lowest in higher vitamin D tertile. DI is index of *β*-cell function after adjustment for insulin resistance, and there was no significant difference of DI among three vitamin D tertiles. This suggested that vitamin D maybe have no influence on compensatory secretion function of *β*-cell.

Our study also demonstrated that men distinguish from women in the relationship of vitamin D with insulin sensitivity and *β*-cell function. In our study population divided by gender, the vitamin D levels of men were not associated with insulin sensitivity and *β*-cell function. Nevertheless, there was significant association between vitamin D and insulin sensitivity and *β*-cell function in women. After adjustment for all kinds of metabolic confounding factors, the association was still significant. Until now, rare studies focused on the gender difference in relationship of vitamin D with insulin sensitivity and *β*-cell function. Lee et al. reported that there was gender difference in the relationship between vitamin D and type 2 diabetes risk among Koreans, and the association was stronger in young women. Gonadal hormone was speculated as an initiating factor; however, further study is needed for elucidating mechanisms [21].

Considering the association between vitamin D and insulin sensitivity and *β*-cell function, vitamin D supplement may be one promising way to improve insulin resistance and reduce risk of diabetes. An intervention study conducted by Mitri et al. suggested that 25-hydroxyvitamin D supplements in dosage of 2000 UI/day can significantly improve islet *β*-cell function [22]. A 6-month randomized controlled trial carried out in south Asian female population demonstrated that 25-hydroxyvitamin D supplements in dosage of 4000 UI/day can improve insulin sensitivity of patients with vitamin D deficiency, but no effects were observed on dyslipidemia [23]. Because of the regional and ethnic differences, until now, no international consensus was reached about the two doses of vitamin D supplement we had regulated. Given the existence of gender differences in the relationship between vitamin D and insulin sensitivity, the supplement of vitamin D may also need to consider difference of gender.

Because the subjects in this study were all citizens in one district and the blood samples were collected in narrow time interval, the geographic and seasonal disturbance on vitamin D was minimized in our study. Of course our study was just a cross sectional study, and we could not affirm the causal relationship between vitamin D and insulin sensitivity and *β*-cell function. In conclusion, our study demonstrated that serum 25-OHD is associated with insulin sensitivity and *β*-cell function for female newly diagnosed type 2 diabetes patients; the association is ambiguous in males. This association may be instructive and meaningful to the supplement of vitamin D and improvement for insulin sensitivity and *β*-cell function.

## Figures and Tables

**Table 1:** The characteristic of three 25-hydroxy vitamin D tertiles [normal distributed data $\overset{-}{X}$ ± *s*, skewed distribution data $\overset{-}{X}$ (95% CI)].

	T1	T2	T3	*F*	*P*
Number	132	132	131		
25-hydroxy vitamin D (nmol/L)	29.15 ± 6.15	42.49 ± 3.78	59.83 ± 8.17	785.862	<0.001
Gender (male/female)	45/87	42/90	70/61	15.473^#^	<0.001
Age (years)	59.37 ± 8.03	58.95 ± 8.12	60.54 ± 8.29	1.339	0.263
BMI (kg/m^2^)	26.10 ± 3.25	26.30 ± 3.21	25.57 ± 3.24	1.805	0.166
SBP (mmHg)	140.29 ± 18.03	140.93 ± 17.45	139.29 ± 17.65	0.286	0.752
DBP (mmHg)	83.04 ± 11.40	82.75 ± 10.58	82.87 ± 10.95	0.022	0.978
TG (mmol/L)	2.28 (2.03, 2.53)	2.30 (2.00, 2.61)	1.90 (1.64, 2.17)^a^	5.584	0.004
TC (mmol/L)	5.32 ± 1.14	5.28 ± 0.95	5.01 ± 0.98^a^	3.476	0.032
HDL (mmol/L)	1.33 ± 0.31	1.26 ± 0.27	1.27 ± 0.26	2.188	0.114
LDL (mmol/L)	3.05 ± 0.83	3.04 ± 0.78	2.89 ± 0.77	1.744	0.176
ALT (U/L)	27.77 (24.26, 31.27)	25.16 (22.63, 27.70)	24.88 (20.90, 28.86)	1.549	0.214
GGT (U/L)	47.96 (39.26, 56.65)	41.34 (36.01, 46.66)	42.18 (35.79, 48.46)	0.740	0.478
FPG (mmol/L)	7.13 ± 1.63	7.25 ± 1.44	7.07 ± 1.37	0.484	0.617
PG120 (mmol/L)	13.95 ± 3.42	13.89 ± 3.38	14.16 ± 3.63	0.796	0.796
HbA1c (%)	6.60 ± 0.96	6.81 ± 1.01	6.67 ± 1.04	0.229	0.229

25-Hydroxy vitamin D tertiles: low tertile T1, middle tertile T2, and high tertile T3. Abnormally distributed continuous variables including TG, ALT, and GGT were log-transformed before analysis; however, nontransformed values were displayed for ease of interpretation. *P*: ANOVA test among three tertiles (*χ* ^2^ test for categorical data). ^a^compared with T1 group. ^#^Statistics was *χ* ^2^ value.

**Table 2:** The comparison of insulin sensitivity and *β*-cell function in total subjects [$\overset{-}{X}$ (95% CI)].

	T1	T2	T3	*P*1	*P*2	*P*3	Trend test *F* value	Trend test *P* value
HOMA-IR	4.64 (4.05, 5.23)	4.32 (3.82, 4.81)	3.63 (3.20, 4.06)^a^	0.019	0.054	0.061	7.438	0.007
Matsuda ISI	2.96 (2.69, 3.24)	3.01 (2.70, 3.33)	3.53 (3.24, 3.81)^a,b^	0.002	0.017	0.058	10.911	0.001
INSR	5.60 (5.01, 6.18)	5.65 (5.08, 6.21)	4.51 (4.02, 5.00)^a,b^	0.013	0.131	0.063	6.019	0.015
DI	13.47 (12.38, 14.55)	14.06 (12.87, 15.26)	13.52 (12.46, 14.57)	0.834	0.815	0.074	0.018	0.893

25-Hydroxy vitamin D tertiles: low tertile T1, middle tertile T2, and high tertile T3. Abnormally distributed continuous variables including HOMA-IR, Matsuda ISI, INSR, and DI were log-transformed before analysis; however, nontransformed values were displayed for ease of interpretation. *P*1: ANOVA test among three tertiles unadjusted for confounding factors. *P*2: adjustment for sex, age, BMI, SBP, and DBP; *P*3: further adjustment for TG, TC, HDL, LDL, ALT, GGT, and HbA1c in addition to *P*2. ^a^compared with T1 group; ^b^compared with T2 group.

**Table 3:** The comparison of insulin sensitivity and *β*-cell function after being divided by gender $[\overset{-}{X}$ (95% CI)].

	T1	T2	T3	*P*1	*P*2	*P*3	Trend test *F* value	Trend test *P* value
Male	52	52	53					
HOMA-IR	4.67 (3.80, 5.54)	4.29 (3.29, 5.29)	3.90 (3.27, 4.54)	0.524	0.764	0.493	1.299	0.256
Matsuda ISI	3.37 (2.77, 3.97)	3.36 (2.81, 3.90)	3.51 (3.13, 3.89)	0.600	0.880	0.786	0.879	0.350
INSR	4.42 (3.40, 5.44)	4.41 (3.54, 5.28)	4.15 (3.56, 4.74)	0.967	0.977	0.920	0.031	0.861
DI	11.73 (9.84, 13.62)	11.77 (10.11, 13.44)	12.27 (11.06, 13.48)	0.558	0.880	0.349	1.159	0.281
Female	79	79	80					
HOMA-IR	4.62 (3.84, 5.41)	4.33 (3.76, 4.89)	3.32 (2.75, 3.90)^ab^	0.011	0.022	0.024	8.264	0.004
Matsuda ISI	2.75 (2.47, 3.03)	2.86 (2.47, 3.24)	3.55 (3.11, 3.99)^a,b^	0.003	0.008	0.014	10.495	0.001
INSR	6.20 (5.51, 6.89)	6.22 (5.52, 6.93)	4.93 (4.11, 5.75)^a,b^	0.014	0.026	0.013	7.084	0.008
DI	14.37 (13.07, 15.67)	15.13 (13.60, 16.67)	14.94 (13.18, 16.71)	0.970	0.937	0.074	0.009	0.923

25-Hydroxy vitamin D tertiles: low tertile T1, middle tertile T2, and high tertile T3. Abnormally distributed continuous variables including HOMA-IR, Matsuda ISI, INSR, and DI were log-transformed before analysis; however, nontransformed values were displayed for ease of interpretation. *P*1: ANOVA test among three tertiles unadjusted for confounding factors. *P*2: adjustment for sex, age, BMI, SBP, and DBP. *P*3: further adjustment for TG, TC, HDL, LDL, ALT, GGT, and HbA1c in addition to *P*2. ^a^compared with T1 group; ^b^compared with T2 group.
